# Electrochemical Sulfite Sensing: Current Trends and Challenges in Food Quality Control and Real Samples

**DOI:** 10.3390/foods15050948

**Published:** 2026-03-07

**Authors:** Arnoldo Vizcarra, Lucas Patricio Hernández-Saravia

**Affiliations:** 1 Instituto de Alta Investigación, Universidad de Tarapacá, Av. Senador Luis Valente Rossi 1501, Arica 1000000, Chile; arnoldo.vizcarra.rojas@gmail.com; 2 Laboratorio de Bionanomateriales, Facultad de Recursos Naturales Renovables, Universidad Arturo Prat, Av. Arturo Prat s/n, Campus Huayquique, Iquique 1100000, Chile

**Keywords:** electrochemical sensing, sulfite determination, food quality control, modified electrodes, food matrices

## Abstract

The analytical surveillance of sulfite species (SO_3_^2−^, SO_2_ and HSO_3_^−^) is critical for food safety due to their roles as preservatives and potent allergens. Despite stringent regulations, conventional methods like Monier-Williams distillation remain limited by labor-intensive protocols and matrix interferences. This review elucidates the chemical mechanisms of sulfites in food matrices and critically evaluates recent advancements in electrochemical sensing. A primary focus is placed on delineating physicochemical bottlenecks, such as electrode fouling and cross-reactivity from polyphenols and organic acids, which hinder commercialization. We analyze the strategic integration of nanostructured interfaces—including bimetallic nanoparticles, carbon-based hybrids (rGO/PPy), and nanozymes—to reduce oxidation overpotentials and enhance sensitivity below regulatory thresholds. Furthermore, the transition from laboratory prototypes to decentralized, field-deployable platforms using screen-printed electrodes (SPEs) and smartphone-based potentiostats is explored. By synthesizing technical innovations with “green” analytical principles, this work provides a roadmap for real-time quality control in the food industry, bridging the gap between fundamental electrochemistry and industrial scalability.

## 1. Introduction

The biochemical significance of sulfiting agents (E220–E228) in agri-food systems has undergone rigorous re-evaluation due to escalating clinical concerns regarding their role in hypersensitivity reactions and respiratory pathologies [1]. As multifaceted preservatives, sulfites exert potent antimicrobial and antibrowning effects, inhibiting both enzymatic (polyphenol oxidase-mediated) and non-enzymatic (Maillard reaction) degradation across diverse food matrices [2,3,4,5]. Consequently, global regulatory frameworks necessitate stringent labeling requirements, typically triggered at a threshold concentration of 10 mg/kg or 10 mg/L (SO_3_^2−^) [6]. This regulatory landscape demands robust analytical surveillance to optimize preservative efficacy while ensuring strict adherence to consumer safety standards and international trade directives.

Despite its necessity, the quantification of sulfite species is inherently complex, primarily due to the chemical instability of the analyte and the intricate nature of food-based “recalcitrant” matrices. Traditional methodologies, such as the Monier-Williams distillation or various titrimetric protocols, are frequently hampered by poor selectivity and high susceptibility to interferents, requiring specialized analytical proficiency and labor-intensive sample preparation [7]. While modern high-throughput instrumental techniques (e.g., High-performance liquid chromatography, ion chromatography, or capillary electrophoresis) offer superior sensitivity, their implementation is often precluded by prohibitive capital expenditure and the requirement for sophisticated infrastructure [8,9]. This creates a technological disparity, particularly for small-to-medium enterprises (SMEs) that lack the fiscal capacity for high-end analytical maintenance.

Electrochemical sensing platforms represent a compelling alternative for decentralized testing, characterized by their inherent scalability, cost-effectiveness, and potential for “point-of-use” application [10,11,12,13]. The fundamental redox thermodynamics of sulfite, which allow for both anodic oxidation and cathodic reduction [14,15], render it a prime candidate for electroanalytical determination. The success of screen-printed electrode (SPE) technologies in clinical diagnostics—most notably in glucose biosensing—serves as a benchmark for the development of disposable, user-friendly analytical tools for food quality control [16,17].

Remarkably, despite the prevalence of colorimetric assays, a commercially viable electrochemical sulfite sensor for food analysis remains elusive. This review critically delineates the physicochemical and engineering bottlenecks that have impeded the commercialization of such devices, while synthesizing recent breakthroughs in electrode modification and nanomaterial integration [18,19]. Furthermore, while the primary focus is directed toward food and beverage matrices, the electrochemical principles discussed herein provide significant cross-disciplinary insights for environmental monitoring, where atmospheric SO_2_ remains a critical pollutant. By evaluating the trajectory from laboratory-scale prototypes to portable diagnostic formats, this work aims to identify the most promising technologies for democratizing high-precision sulfite analysis in the food industry.

## 2. Preservative Efficacy and Antioxidant Mechanisms of Sulfites

Sulfites serve as pivotal multifunctional additives in food science, primarily valued for their potent reducing capabilities. In synergistic action with ascorbates, sulfites constitute a robust antioxidant defense system that mitigates oxidative degradation in food and beverage systems exposed to atmospheric oxygen [3]. Their application spans a diverse range of commodities, including processed meats, viticultural products, malt beverages, and various horticultural derivatives such as pomaceous fruit juices, preserves, and dehydrated fruits [2,20]. The steady-state concentration of sulfite species is highly dependent on the intrinsic physicochemical properties of the food matrix and the specific parameters of industrial processing. In pre-packaged, minimally processed fruits, vegetables, and crustaceans, sulfites are indispensable for extending shelf life by inhibiting chromogenic pathways that result in deleterious browning, which would otherwise compromise consumer acceptability [10,21,22,23].

### 2.1. Inhibition of Enzymatic Browning: Polyphenol Oxidase (PPO) Kinetics

The biochemical deterioration of plant-based products is largely governed by the activity of polyphenol oxidase (PPO) [24]. The catalytic mechanism involves the oxygen-mediated hydroxylation and subsequent oxidation of phenolic derivatives (I) into highly reactive o-quinone intermediates (II). These quinones initiate a complex polymerization cascade leading to the formation of melanins and other undesirable pigmented polymers.

**Figure 1:**
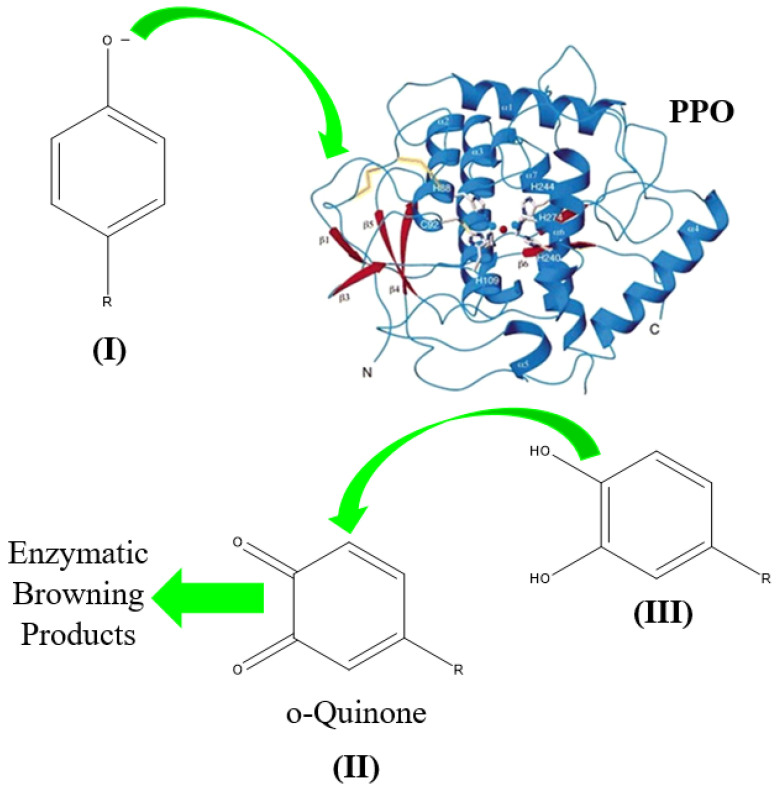
Enzymatic browning reaction scheme.

Sulfite exerts a dual inhibitory effect on this pathway: it acts as a competitive inhibitor of the PPO enzyme and functions as a reducing agent that reconverts o-quinones back to their more stable 1,2-dihydroxybenzene precursors (III). By intercepting the reaction at these nascent stages, sulfites preserve the polyphenolic profile of the matrix (see Figure 1). This is particularly critical in enology, where polyphenols are fundamental to the organoleptic architecture (flavor, astringency, and color) and the purported cardiovascular health benefits of the final product [25].

### 2.2. Mitigation of Non-Enzymatic Browning: The Maillard Reaction

**Figure 2:**
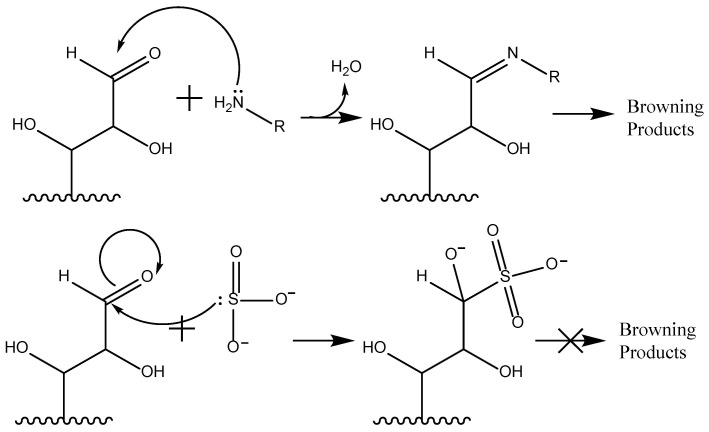
Reaction schemes highlighting the onset of non-enzymatic browning and the preservative action of sulfite.

Beyond enzymatic control, the nucleophilic properties of the sulfite anion play a decisive role in inhibiting non-enzymatic Maillard browning [5,26,27,28]. The Maillard cascade begins with the nucleophilic attack of amino groups (derived from free amino acids or proteins) on the carbonyl carbon of reducing sugars, yielding N-substituted glycosylamines (Figure 2). Subsequent Amadori rearrangements and fragmentations produce advanced glycation end-products (AGEs) and nitrogenous polymers (melanoidins). While these reactions are desirable for flavor development in baked goods, they often signal bitterness and spoilage in raw tissues and proteins.

Sulfite additives effectively quench this pathway by participating in nucleophilic addition to carbonyl functionalities. This competitive addition sequesters the reactive carbonyl sites, preventing amine condensation at the source. Due to the high nucleophilicity of the sulfite anion, this reaction proceeds efficiently across a broad pH range without the requirement for acid or base catalysis [29,30].

### 2.3. Sulfite-Disulfide Interactions and Rheological Implications

Furthermore, sulfites interact dynamically with the proteinaceous fraction of food through the cleavage of disulfide bonds (RSSR). This sulfitolysis reaction results in the formation of a free sulfhydryl group (RSH) and an S-sulfonate (RSSO_3_^−^) [31]. This chemical modification is extensively leveraged in cereal chemistry, specifically in dough conditioning. By selectively weakening the gluten network through disulfide reduction, sulfites optimize the rheological properties of dough, enhancing extensibility and consistency prior to thermal processing [32]. It is essential to note that within these complex systems, sulfites exist in a dynamic equilibrium between free and reversibly bound forms, influenced by the specific nucleophilic environment of the food matrix.

## 3. Clinical Significance and Metabolic Implications of SO_3_^2−^ Ingestion

The pathophysiological consequences of SO_3_^2−^ inhalation are well-documented, with extensive literature linking atmospheric exposure to occupational and environmental pulmonary pathologies [33,34]. SO_3_^2−^ is known to induce significant airway inflammation via neutrophil activation, directly contributing to bronchoconstriction and the exacerbation of asthmatic phenotypes [35]. These clinical concerns have prompted regulatory bodies to implement stringent labeling requirements for sulfite additives in food and beverage products. Despite this, contemporary clinical trials evaluating asthmatic susceptibility to sulfites in viticultural products have yet to definitively elucidate the specific molecular triggers responsible for the sulfite-induced asthmatic response [1].

Current evidence suggests that the biochemical mechanism involves sulfite-mediated neutrophil activation, characterized by the accelerated release of ROS—predominantly hydrogen peroxide (H_2_O_2_)—and chemotactic factors such as interleukin-8 (IL-8) [36,37]. Interestingly, neutrophils of both human and murine origin have been observed to generate endogenous sulfite spontaneously upon stimulation by bacterial endotoxins. This suggests that the sulfite anion may serve a functional role in mediating antimicrobial and pro-inflammatory cascades [35].

Beyond its role as an exogenous food additive, sulfite is a constituent of endogenous metabolism, primarily generated through the catabolism of sulfur-containing amino acids. Mammalian systems utilize sulfite oxidase (EC 1.8.3.1) to catalyze the conversion of sulfite into the less toxic sulfate (SO_4_^2−^), thereby maintaining systemic homeostasis [38,39,40]. In healthy adults, basal plasma sulfite concentrations are highly modulated by dietary intake and lifestyle, typically ranging between 0.1 and 10 µM [41]. Research indicates that basal levels (averaging 0.4 to 1.210 µM) can undergo a ten-fold transient increase approximately one-hour post-ingestion of red wine (e.g., 200 mL containing 320 mg of SO_3_^2−^), reflecting the kinetics of oral absorption and subsequent metabolic clearance [42]. While excessive ingestion can acutely elevate plasma concentrations beyond 100 µM, the human organism typically exhibits rapid clearance mechanisms to restore homeostatic levels [38]. Although sustained elevations have been documented in patients with renal impairment, it remains ambiguous whether these levels contribute to disease progression or are merely biomarkers of reduced metabolic clearance. Notably, massive oral boluses of sulfite in healthy subjects have not consistently resulted in significant adverse clinical outcomes. Furthermore, recent in vitro and in vivo investigations suggest a more nuanced physiological role for the sulfite anion; studies have demonstrated its capacity to inhibit lipid peroxidation, indicating that at regulated concentrations, sulfite may actually confer protective benefits against oxidative stress-mediated cellular damage [43].

## 4. Sulfite Measurement

**Figure 3:**
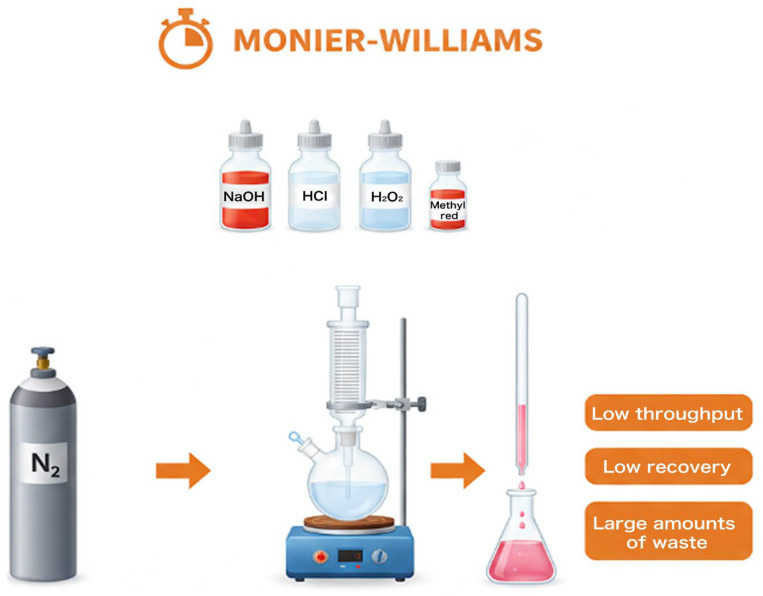
Illustration of the Monier-Williams method. Adapted with permission from Ref. [7].

The rigorous quantification of sulfite species is a fundamental requirement for regulatory compliance and food quality assurance. Historically, the Monier-Williams method (illustrated in Figure 3), adopted as the official reference by the Association of Official Analytical Chemists (AOAC), remains the benchmark [44]. This technique involves the acid-catalyzed release of SO_2_ from the food matrix under reflux conditions, typically utilizing 0.5 M HCl, as described by Equation (1):

(1) $${SO}_{3}^{2-}+{2H}^{+}\to {SO}_{2(g)}+H_{2}O\;$$

The liberated SO_2_ is swept by an inert nitrogen carrier gas into a 3% H_2_O_2_ trap. The subsequent in situ oxidation of SO_2_ generates sulfuric acid, as described by Equation (2):

(2) $${SO}_{2(g)}+H_{2}O_{2}\to H_{2}{SO}_{4}\;$$

The resulting acidity is quantified via titrimetric analysis with a standardized base. While the Monier-Williams procedure is valued for its low capital expenditure and reliance on standard laboratory glassware, it is characterized by significant throughput limitations and a lack of sensitivity for trace-level detection. Furthermore, the method is susceptible to false-positive results due to the co-distillation of volatile organic acids, which can lead to an overestimation of the total sulfite content [45].

### 4.1. Instrumental Refinements and Chromatographic Techniques

**Figure 4:**
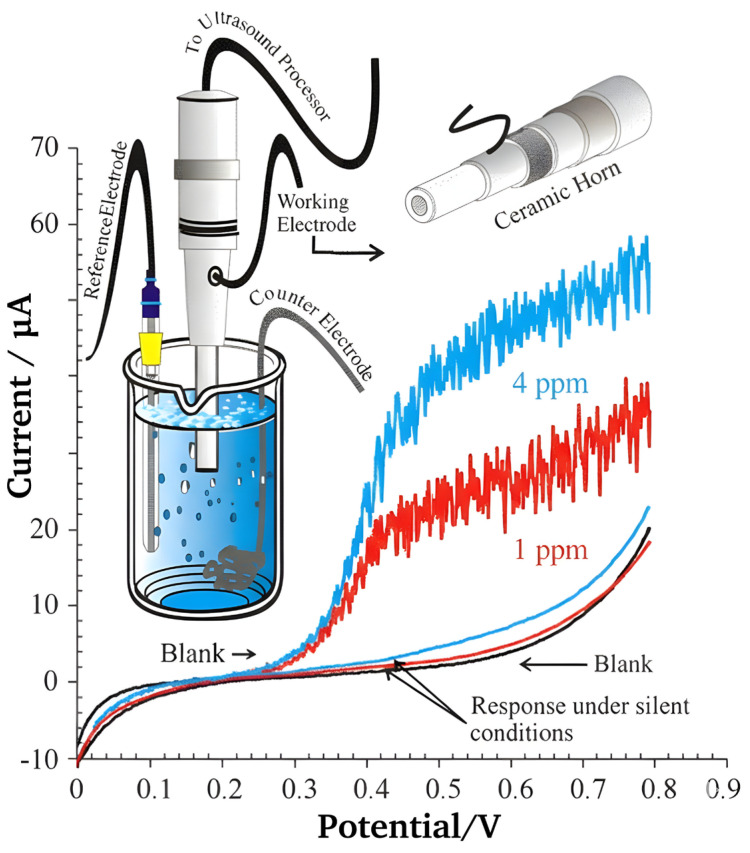
Illustration of the electrochemical method. Adapted with permission from Ref. [32].

To overcome the selectivity issues of classical titration, various analytical modifications have been proposed. Iodometric titrations provide a more specific redox-based alternative, as they are largely immune to the interference of volatile acids [46]. More advanced instrumental couplings include ion chromatography (IC) [9,47,48] and capillary electrophoresis (CE) [7,49,50] for the direct measurement of the sulfate byproduct. Although these methods effectively eliminate chemical interferences, they necessitate substantial investment in specialized instrumentation. Electrochemical detection (ECD) [51] has emerged as a robust alternative, offering quantification via differential pulse voltammetry [10,52,53], polarography [54] (see Figure 4), cyclic voltammetry [55,56,57], square wave voltammetry [58,59,60], potentiometry [61], or amperometry [6,62,63]. Amperometric detection, particularly when integrated into post-column ion chromatography systems, is widely regarded as a superior approach for routine analysis. This configuration allows for the direct processing of liquid samples, minimizes labor-intensive sample preparation, and circumvents the time-consuming distillation step. The synergy between chromatographic resolution and the high sensitivity of electrode assemblies provides a lower limit of detection (LOD) compared to traditional volumetry.

### 4.2. Flow Injection Analysis (FIA) and Gas Diffusion Membranes

**Figure 5:**
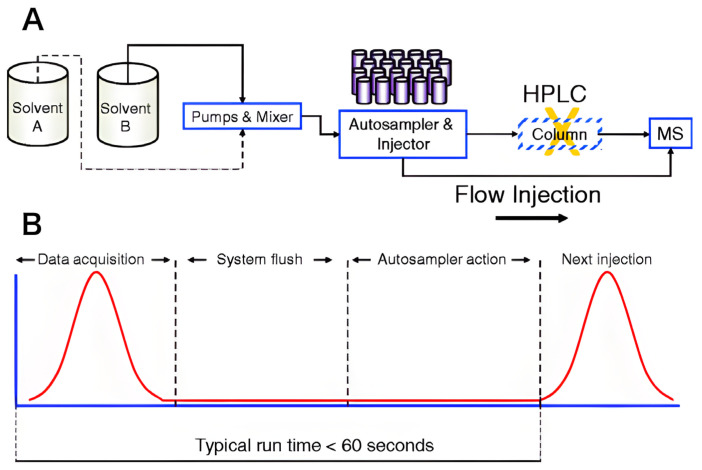
Illustration of FIA-mass spectrometry (MS) (**A**) employing widely available autosamplers that have been originally designed for HPLC (**B**). Reproduced from [65].

In recent years, Flow Injection Analysis (FIA) [64] has gained predominance over conventional Liquid Chromatography (LC) for high-throughput sulfite monitoring (see Figure 5) [7,65,66]. Rather than relying solely on column-based separation, FIA systems exploit the intrinsic volatility of SO_3_^2−^ through gas-diffusion modules. These dual-channel systems utilize hydrophobic membranes (typically silicone or PTFE) to isolate the analyte from the complex sample matrix [67,68]. Upon acidification of the donor stream, the generated SO_3_^2−^ permeates the membrane into an acceptor stream, where it is subsequently quantified using amperometric [69] or potentiometric [70] sensors. While FIA offers high precision and automation, these systems—along with most modern instrumental methods—require significant technical expertise and maintenance. Consequently, there is an ongoing shift in food analytical chemistry toward developing simplified, cost-effective sensing platforms that can achieve comparable selectivity and sensitivity without the infrastructural burden of sophisticated flow-based architectures.

### 4.3. Modified Electrodes for Sulfite Detection in Complex Matrices Such as Food and Water

The primary objective in developing advanced electrochemical platforms for SO_3_^2−^ quantification is the strategic reduction in the overpotential required to initiate analyte oxidation [8]. By shifting the onset potential toward more cathodic regions, the contribution of ubiquitous interfering species—most notably L-ascorbic acid and various polyphenolic compounds—to the faradaic signal can be significantly attenuated. Current research paradigms generally bifurcate into the deployment of nanoparticles, transition metal complexes or bio-catalytic agents, with the former predominating due to their robust chemical stability and tunable redox properties.

A diverse array of electroactive mediators has been evaluated to facilitate this electron transfer [51]. Notable examples include transition metal hexacyanoferrate thin films (e.g., Co, Lu, and Ni) [69,71,72,73], ferrocene derivatives [74], and complexes [6]. While these mediators were historically employed as homogeneous solution-phase species, contemporary trends favor their immobilization onto electrode surfaces as mono- or multilayer films, or their integration into the bulk of composite architectures, such as sol–gel matrices, to enhance durability and sensor longevity.

Despite extensive fundamental studies characterizing the redox transitions and charge-transfer kinetics of these modified surfaces, a significant gap remains regarding their systematic validation in complex food and environmental matrices. Although these modifications invariably augment the sensitivity toward sulfite, their selectivity over co-existing reductants remains a critical bottleneck. Specifically, since the formal potentials of most synthetic complexes remain more positive than that of ascorbate oxidation, rigorous sample pre-treatment or chromatographic separation (e.g., FIA or HPLC with electrochemical detection) remains essential to mitigate matrix effects.

Finally, the practical deployment of these technologies must balance analytical sophistication with operational simplicity. While elaborate organometallic complexes [66] offer high theoretical performance, the electrodeposition of simple metallic or metal oxide nanoparticles (such as Cu, Ag, Au, or Pt) [21,55,56,75] onto carbonaceous substrates provides a robust and accessible alternative. These nanostructured interfaces often yield comparable enhancements in detector sensitivity and stability, presenting a more feasible pathway for high-throughput quality control in the food industry by non-specialist personnel.

## 5. Electrochemical Sensors Using Voltammetric Methodologies

The application of voltammetric techniques is pivotal for the precise quantification of sulfite species in food matrices, offering a robust equilibrium between mechanistic insight and high-sensitivity detection. Cyclic Voltammetry (CV) serves as the primary diagnostic tool for characterizing the electrocatalytic interaction between the modified electrode surface and the sulfite ions. By analyzing peak potential (E_p_) and peak current (I_p_) variations as a function of the square root of the scan rate (ν^1/2^), the kinetic regime—whether diffusion-controlled or adsorption-controlled—can be elucidated. This fundamental characterization is essential for evaluating the efficiency of mediators, such as metal complexes or oxides, in lowering the overpotential for the SO_3_^2−^ to SO_4_^2−^ conversion.

To further validate the proposed electrochemical approach, its analytical performance was benchmarked against the Monier-Williams (AOAC official method) and High-performance liquid chromatography (HPLC). As summarized in Table 1, the electrochemical method demonstrates superior operational efficiency, particularly regarding analysis time and portability.

While CV is indispensable for fundamental characterization, Differential Pulse Voltammetry (DPV) is the preferred methodology for analytical validation in complex food matrices. The inherent advantage of DPV lies in its ability to sample the current immediately before the end of each potential pulse, which effectively discriminates the faradaic current from the background charging (capacitive) current. This discrimination results in a significantly lower limit of detection (LOD) compared to linear sweep techniques, making it suitable for monitoring sulfite levels below the regulatory thresholds established by organizations like the FDA, as can be seen in Table 2.

The success of these voltammetric methodologies in “real-world” samples—such as enological matrices or aqueous food extracts—depends heavily on the optimization of pulse parameters, including pulse amplitude, width, and increment. In samples like white wine, where L-ascorbic acid often acts as a significant interferent, DPV provides the superior resolution necessary to distinguish the sulfite oxidation peak from overlapping signals. Furthermore, the integration of DPV with modified carbon-based electrodes allows for high repeatability and sensitivity, even in the presence of surface-active proteins or polyphenols that typically challenge the stability of the electrochemical interface.

**Figure 6:**
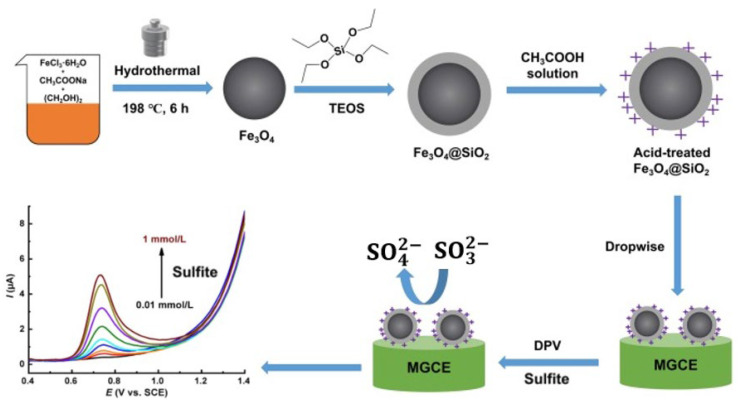
Schematic diagram of Fe_3_O_4_@SiO_2_(acid-treated)/MGCE preparation and the sulfite electrochemical detection. Adapted with permission from Ref. [78].

Zhang et al. (2024) [78] developed a simple electrochemical platform for the detection of nitrite and sulfite in milk samples. The substrate was a Magnetic Glassy Carbon Electrode (MGCE), which was modified with acid-treated Fe_3_O_4_@SiO_2_ nanoparticles to form the modified electrode (shown in Figure 6). The acid treatment was a key step to introduce positive charges, enhancing the electrostatic attraction between the sensor surface and the anionic analytes. Using DPV and CV as analytical Methods, the sensor provided an LOD of 31.57 µM for sulfite. The repeatability and reproducibility were reflected in recoveries ranging from 85.18% to 111.02% with an RSD of 0.23–4.80%. While the magnetic properties of the MGCE facilitate easy electrode preparation and potential sample cleanup, the sensitivity for sulfite (LOD of 31.57 µM) is significantly lower—by several orders of magnitude—than that of the rGO/PPy NTs or Ce^3+^-doped CuO systems. This suggests that while electrostatic attraction helps, the inherent catalytic activity of silica-coated iron oxide is inferior to that of metal oxides or conducting polymer hybrids for sulfite oxidation.

Jahani et al. (2022) [79] introduced a powerful sensing strategy using a Graphite Screen Printed Electrode (GSPE) modified with a reduced graphene oxide/polypyrrole nanotubes (rGO/PPy NTs) nanocomposite. This modified electrode (rGO/PPy NTs-GSPE) was synthesized via a hydrothermal protocol and used for the detection of sulfite in food samples using DPV as the main quantitative Method. The sensor exhibited an exceptional linear range of 0.04 to 565.0 µM, an extremely low LOD of 0.01 µM, and a sensitivity of 0.0483 µA/µM. The structural morphology of PPy nanotubes combined with the high surface area of rGO provides a superior electronic environment for sulfite oxidation, as evidenced by the lowest LOD among the analyzed works. This highlights the advantage of 1D/2D hybrid nanomaterials in achieving “ultrasensitive” detection, making it highly applicable for trace analysis in complex matrices where higher sensitivity is required to overcome matrix effects.

Liu et al. (2022) [80] fabricated a nonenzymatic sensor using GCE modified with a chitin/graphene oxide (chitin/GO) nanocomposite. The modified electrode leverages chitin, a polysaccharide isolated from wild morel mushrooms, to improve the dispersion and biocompatibility of the GO layer. The sensor was characterized using DPV and applied to the determination of sulfite in food samples as the Method Sample. It achieved a linear range of 1 to 960 µM, showing performance comparable to or exceeding other carbon-based sensors. The sensor achieved an LOD of 0.021 µM and a sensitivity of 0.02751 μA mM^−1^. Its feasibility was confirmed through the analysis of vinegar and pickle water samples. The use of chitin as a “green” modifier provides a sustainable alternative to synthetic polymers like Nafion or PPY. However, the study’s reliance on GO implies a lower conductivity compared to rGO, which might explain the slightly higher LOD compared to more advanced hybrid nanostructures, though the synergistic effect with chitin effectively facilitates charge transfer.

Malakootian et al. (2022) [22] reported an efficient electrochemical sensor based on a SPE substrate modified with a Ce^3+^-doped CuO nanocomposite. The modified electrode (Ce^3+^-doped CuO/SPE) was fabricated and characterized using SEM, XRD, and EDX to confirm its morphology and composition. Using DPV as the primary analytical Method, the sensor targeted sulfite ions in water and soft drinks as the Method Sample. The analytical performance revealed a wide linear range from 0.6 to 400 µM and a low LOD of 0.08 µM. The use of rare-earth doping (Ce^3+^) in transition metal oxides is a strategic choice to enhance oxygen vacancy density and catalytic sites; however, while the SPE substrate facilitates portability and low cost, the irreversible and diffusion-controlled nature of the sulfite oxidation at this surface may lead to electrode fouling over extended use, requiring disposable strategies rather than long-term stability.

Luo et al. (2021) [81] developed a sophisticated electrochemical sensor for sulfite. The substrate utilized was a GCE, which was functionalized to create a modified electrode through the one-step electrodeposition of Pt-Pd bimetallic nanoparticles onto a chitosan/nitrogen-doped graphene (N-Gra) matrix. The Method employed for quantitative analysis was DPV, which provided sufficient electrochemical resolution to separate the oxidative peaks of the three analytes. For sulfite, the sensor achieved a linear range of 8–600 µM with a LOD of 5.5 µM. The Method Sample application was demonstrated in pharmaceuticals, specifically Vitamin C injections, to monitor drug quality and preservatives. The integration of bimetallic Pt-Pd nanoparticles significantly lowers the oxidation overpotential compared to monometallic systems, although the reported LOD for sulfite (5.5 µM) is notably higher than that of more specialized sulfite sensors (e.g., 0.01 µM for rGO/PPy systems), suggesting that this platform prioritizes multiplexing capabilities over extreme sensitivity for individual ions.

**Figure 7:**
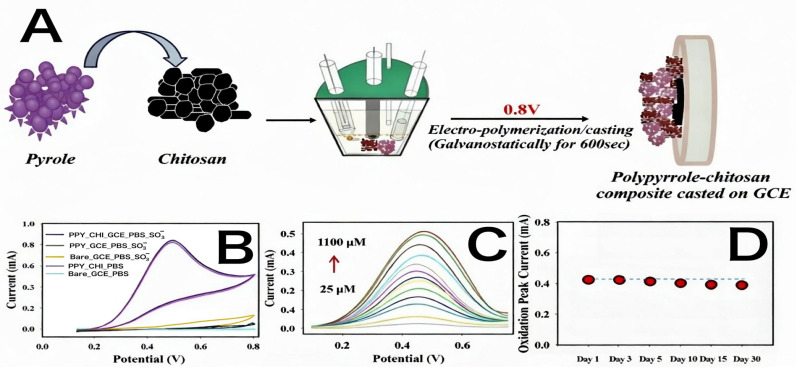
Schematic illustration of synthetic procedure for electropolymerization of polypyrrole-chitosan (**A**), Cyclic voltammograms obtained for PPY-CHI film modified GCE and bare GCE in a 0.1 M PBS (pH 8.5) with and without 1.5 mM sulfite (**B**), Differential pulse voltammogram obtained for PPY-CHI film modified GCE towards increment in sulfite concentration (**C**) and Peak current of PPY-CHI/GCE in 800 μM sulfite recorded from the first till one month (**D**). Reproduced from [82].

Sudha et al. (2021) [82] explored a GCE modified with copper-based metal oxide nanoparticles (Cu_2_O, CuO, and CuNa_2_(OH)_4_). Among these, the CuO-modified electrode showed the best performance for sulfite oxidation in commercial wine samples. The linear range was 0.2 to 15 mM with an LOD of 1.8 μM and a sensitivity of 48.5 μA mM^−1^ cm^−2^. The work demonstrates how tuning the crystal structure (monoclinic CuO vs. cubic Cu_2_O) directly impacts catalytic efficiency, show Figure 7. While copper is an abundant and cheap catalyst, its LOD is an order of magnitude higher than gold or La^3+^-doped sensors, making it more suitable for samples with higher sulfite concentrations, like industrial wines, rather than trace analysis.

Adeosun et al. (2020) [83] reported the synthesis of a conductive polypyrrole-chitosan (PPY-CHI) thin film on a GCE. The modified electrode displayed high electrocatalytic activity for sulfite oxidation using DPV. It achieved a linear range of 50 to 1100 μM, an LOD of 0.21 μM, and a sensitivity of 15.28 μA μM^−1^ cm^−2^. The sensor was noted for its repeatability and reproducibility and was applied to food and biological samples. This study highlights the use of conducting polymers, which offer better biocompatibility for biological samples compared to metal oxides. The sensitivity is lower than that of AuNP-based composites, but the thin-film stability and anti-fouling properties of chitosan/PPY are superior for long-term use.

Zabihpour et al. (2020) [84] introduced an ultrasensitive sensor based on a CPE modified with MgO/SWCNTs and the ionic liquid [Bmim][Tf2N]. The modified electrode was used for the determination of ferulic acid in the presence of sulfite in food samples. The sensor presented a linear range of 0.1–450 µM and a low LOD of 50 nM for sulfite. The practical applicability was demonstrated in red wine and other food-related matrices. The incorporation of an ionic liquid into the carbon paste matrix significantly improves the conductivity and facilitates electron transfer. This multi-component approach (nanoparticles, nanotubes, and ionic liquid) represents the current trend in maximizing sensitivity, although the reproducibility of such complex pastes can be more challenging than fixed film electrodes.

**Figure 8:**
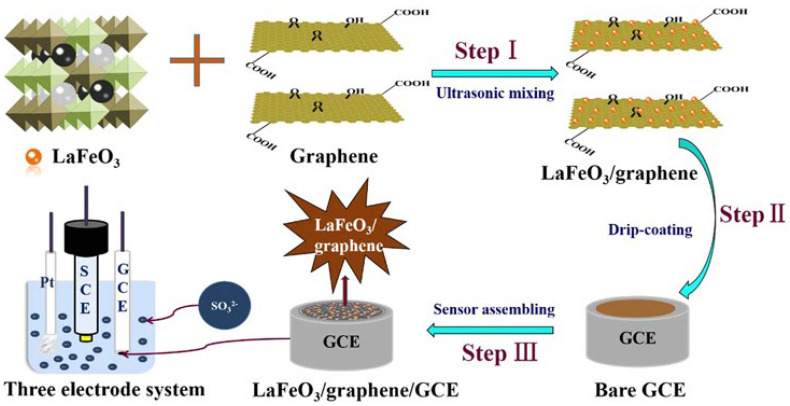
Schematic diagram for the process of fabrication and SO_3_^2−^ test of LaFeO_3_/graphene. Reproduced from [85].

Zhu et al. (2020) [85] designed a sensor using a GCE modified with a LaFeO_3_/graphene composite (see Figure 8). The modified electrode was prepared using a sol–gel method of LaFeO_3_, followed by ultrasonic dispersion with graphene. Using DPV, the sensor achieved a linear range of 1–200 µM with an LOD of 0.21 µM (S/N = 3). This perovskite-graphene hybrid explores the high catalytic activity of LaFeO_3_. While the linear range is narrower than that of the Co_3_O_4_ or AuNP-based sensors, the selectivity in complex matrices is often superior with perovskite structures, although the synthesis process is more complex. The sensor was successfully applied to white wine samples, yielding recoveries between 97.63% and 103.02%.

Beitollahi et al. (2019) [86] utilized a SPE modified with La^3+^-doped Co_3_O_4_ nanocubes. The modified electrode showed electrocatalytic activity in PBS (pH 7.0) using voltammetry. The linear range was 0.7 to 1000.0 µM, with an LOD of 0.090 µM. The sensor demonstrated outstanding recovery in real samples. The choice of an SPE substrate is highly oriented toward commercialization and field use. The use of a rare-earth doped transition metal oxide (La^3+^-Co_3_O_4_) offers a more cost-effective alternative to gold-based sensors while maintaining competitive detection limits in neutral pH.

Manikandan et al. (2018) [87] developed a nanoporous gold microelectrode (NPG-ME) substrate synthesized via electrochemical alloying/dealloying. This modified electrode was applied for the detection of sulfite. The alloying process increased the electrochemically active surface area (ECSA) by 25 times. Using DPV, the sensor showed well-separated oxidation peaks. For sulfite detection, the NPG/µE displayed a broad linear range (5.0–4000 µM) and an LOD of 0.337 µM. The sensor was successfully applied to complex matrices, including water, wine, apple cider, beer, and beef. The use of a microelectrode format is highly beneficial for small-volume samples and potentially for in situ analysis. The nanoporous structure inherently provides a high density of active sites without needing external modifiers like carbon nanotubes, though the long-term mechanical stability of dealloyed structures can be a concern in continuous flow systems.

Sudha et al. (2018) [87] constructed a GCE modified with acid-functionalized multi-walled carbon nanotubes (HOOC-MWCNT) for the simultaneous sensing of sulfite and nitrite. The modified electrode utilized the discrete tube-like morphology and high surface area of HOOC-MWCNT to enhance the signal. The methods used were CV and DPV under neutral conditions. A significant peak separation of 420 mV (CV) between the two anions allowed for simultaneous detection. Under neutral conditions, the sensor allowed for the simultaneous estimation of sulfite and nitrite. DPV analysis yielded a sensitivity of 25.29 μA mM^−1^ cm^−2^ and an LOD of 215 nM for SO_3_^2−^. The operation under neutral conditions is a major practical advantage over acidic-dependent sensors, as it simplifies sample preparation for environmental and food monitoring. However, without the addition of metal nanoparticles, the catalytic currents may be lower, potentially affecting the sensitivity compared to hybrid metal-carbon systems.

Wu et al. (2018) [88] fabricated a nonenzymatic sensor based on a GCE modified with CTAB, chitosan (Chit), and CNT. This modified electrode was designed for the determination of hydroxymethanesulfinate (HMS) in the presence of sulfite in food samples. Using DPV, the sensor achieved an expanded peak-to-peak separation between HMS and sulfite due to the synergetic effect of the CTAB micelles and the CNT-Chit matrix. The integration of a surfactant (CTAB) with a biopolymer (chitosan) successfully addresses the common issue of CNT agglomeration. By focusing on the separation of HMS and sulfite, this work provides a higher degree of analytical selectivity than standard sulfite sensors, which is crucial for identifying illegally added food substances.

Yu et al. (2017) [89] developed a highly sensitive electrochemical sensor for sulfite detection using a GCE as the substrate, modified with gold nanoparticles-reduced graphene oxide (AuNPs-rGO) nano-composites. The modified electrode (AuNPs-rGO/GCE) was fabricated via a dropping method after synthesizing the composite through a chemical co-reduction strategy. Using amperometry at 0.40 V (vs. SCE) in 0.10 M H_2_SO_4_, the sensor achieved a wide linear range of 2.0 × 10^−7^ to 2.3 × 10^−3^ M with a LOD of 4.5 × 10^−8^ M (S/N = 3). The sensitivity was reported as 1026.5 µA mM^−1^ cm^−2^. The study attributes this performance to the synergistic effect between AuNPs and rGO, which enhances the electrocatalytic activity for sulfite oxidation. While the sensitivity is remarkably high compared to other carbon-based sensors, the reliance on a strongly acidic medium may limit its direct applicability in complex food matrices without significant sample pretreatment. The use of amperometry provides excellent sensitivity but is generally more prone to interference from other electroactive species compared to pulse techniques like DPV.

**Table 2:** Electrode materials for sulfite detection by cyclic voltammetry or differential pulse voltammetry: fabrication method, analysed sample, and analytical features (linear range, LOD, LOQ and sensitivity).

Electrode/Substrate	Method	Sample	Linear Range (μM)	LOD (μM)	LOQ (μM)	Sensitivity	Ref.
ZIF-L (Zn)/SPGE	Ultrasonication/Hydrothermal	Well water and tap water	0.04–900.0	0.01	—	0.0198 µA µM^−1^	[52]
CuO/SiO_2_@CPE	Dissolution, stirring, evaporation, drying, and calcination	Tap water	500–5000	1.24	—	7.0 μA mM^−1^	[90]
NPG/GCE	Hydrothermal	Mike, pickle, red wine and tapwater	50–5000	5.12	—	71.76 μA mM^−1^ cm^−2^	[10]
ED-Cu(I)	Electrodeposited	—	160–25,000	0.049	—	14 μA mM^−1^	[55]
Fe_3_O_4_@SiO_2_(acid-treated)/MGCE	Sol–gel and hydrothermal	Milk	0–8000	31.57	—	0.066 μA mM^−1^	[78]
CeO_2_ NPs/SPCE	Ultrasonicated	River water and tap water	0.08–870.0	0.023	0.077	0.043 µA µM^−1^	[91]
AgNPs/PPy@PEDOT:PSS/SLT	Electrodeposited	Milk and rice flour	1–130 ng mL^−1^	0.29 ng g^−1^	—	1.510 μA ng^−1^ mL	[21]
N-HCSs/GCE	Hydrothermal/calcination	Well water and tap water	1.0–100.0	0.35	—	0.0917 µA µM^−1^	[92]
CuMS/GCE	Hydrothermal	Wine	100–15,000	1.26 nM	—	65.42 μA mM^−1^ cm^−2^	[93]
NBC180	Hydrothermal	White wine	0–3000	43	—	0.133 μA mM^−1^ cm^−2^	[94]
rGO/PPy NT-GSPE	Sonication/Hydrothermal/Sonication	River water, well water and apple juice	0.04–565.0	0.01	—	0.0483 μA mM^−1^	[79]
POBI/CS/GCE	Solvothermal	Tap water and artificial lake water	50–900	16.50	—	0.0040 μA mM^−1^	[95]
*p*-ADPA-4-ATP-Au/GC	Electrodeposition/electropolymerization	Biscuit and soup	5.0–160	1.5	5	0.0176 μA mM^−1^	[56]
Graphene printed electrode	^—^	Wine	2–60 mg L^−1^	1.5 mg L^−1^	—	0.65 μA mg L^−1^	[96]
chitin/GO/GCE	Ultrasonicated	Vinegar and pickle water	1–960	0.021	—	0.02751 µA µM^−1^	[80]
Ce^3+^-doped CuO/SPE	Chemical precipitation/hydrothermal	Non-alcoholic malt drink, Orange juice, bottled water and tap water	0.6–400	0.08	—	0.0934 µA µM^−1^	[22]
2FTNE/Co-Ce-NPs/CPE	Sonochemical	Drinking water, tap water and river water	0.07–440.0	0.03	—	0.0854 µA µM^−1^	[97]
MWCNT/IL/AM	Sonochemical	^—^	0–50 × 10^3^	1.6 × 10^3^	—	0.41192 μA mM^−1^	[98]
Tl-Mn_3_O_4_/IL/CPE	Hydrothermal	Drinking water, tap water and river water	0.03–235.0	10.0 nM	—	0.1009 µA µM^−1^	[99]
Pt- Pd NPs/N-Gra/GCE	Hydrothermal	Vitamin C	8–600	5.5	—	0.0267 µA µM^−1^	[81]
PBIF/CS/GCE	Hydrothermal	Tap water	10–200	4.03	—	0.71 µA µM^−1^	[100]
PANI Electrode	Electropolymerization	Red wine and white wine	0–20 ppm	6 × 10^−3^ ppm	—	0.9802 µA ppm^−1^	[57]
GCE/GONRs–AuNPs	Hydrothermal/electrodeposition	Spring water and lake water	10–10,000	1.1	—	266 μA mM^−1^ cm^−2^	[101]
CuO/GCE	Hydrothermal	Wine	5–15 × 10^3^	1.8	—	48.5 μA mM^−1^ cm^−2^	[82]
PPY-CHI/GCE	Electropolymerization	Orange juice, malt drink and human serum	50–1100	0.21	0.7	15.28 μA μM^−1^ cm^−2^	[83]
MTTiPNiH	Hydrothermal	^—^	80–900	30	—	3.97 mA mM^−1^	[102]
MoS_2_/NF/GCE	Hydrothermal	Tap water	5–500	3.3	—	6.784 μA mM^−1^	[103]
MgO/SWCNTs-[Bmim][Tf2N]-CPE	Hydrothermal/solvothermal	Red wine and rice white	0.1–450	50 nM	—	0.2749 μA μM^−1^	[84]
LaFeO_3_/Graphene	Ultrasonicated	White wine	1–200	0.21	—	0.119 μA μM^−1^	[85]
AuNP/TPyP hybrid/FTO	Hydrothermal	—	0–450	—	—	12.8 × 10^–2^ μA μM^−1^	[104]
AgNPs@PANI/rGO/GCE	Sonication/Hydrothermal/Sonication	Drinking water, Tap water and River water	2.7–24.4	77 nM	25.6	128 μA μM^−1^ cm^−2^	[105]
ZTTiPNiH	Hydrothermal	^—^	50–800	50	^—^	14.42 mA mM^−1^	[71]
La^3+^-doped Co_3_O_4_ nanocubes/SPE	Hydrothermal	Tap water, River water and Waste water	0.7–1000.0	0.09	0.29	0.0481 μA μM^−1^	[86]
DP	—	Wine	–	—	—	—	[54]
Pt	—	Apple cider, vinegar and Brown sugar	0–1 g L^−1^	1.908 mg L^−1^	9.60 mg L^−1^	599.79 μA g^−1^ L	[106]
NPG/µE	Electrodeposition	Chile wine, Australian wine, South African wine and Apple cider beer	5 μM–4 mM	3.37 × 10^−7^ M	—	0.011 μA μM^−1^	[87]
HOOC-MWCNT	—	Underground water and kollankalliamman temple pond water	0.5 mM–1.2 mM	215 nM	—	25.29 μA mM^−1^ cm^−2^	[107]
CPE/NiO-NPs/AF	Hydrothermal	Weak liquor, Weak liquor, Well water, Tap water and River water	0.005–500	0.001	—	0.0535 μA μM^−1^	[74]
LuHCF/poly(taurine)/GCE	Electrodeposition	Tap water and the purified drinking water	0–10,000	1.33	—	448 μA mM^−1^ cm^−2^	[72]
GC/SSG-Au	Electrodeposition	—	100–800	—	—	0.028 μA μM^−1^	[88]
GC	—	Red wine	0.2–1.0	0.16	0.534	5.37 μA mM^−1^ cm^−2^	[108]

**ZIF-L (Zn):** Zeolitic imidazolate framework-L (Zn); **CuO/SiO₂:** copper nanoparticles in silica oxide; **NPG:** Nanoporous gold; **ED-Cu(I):** electrodeposited copper nanoparticles; **Fe_3_O_4_@SiO_2_@(acid-treated)/MGCE:** Fe_3_O_4_@SiO_2_ modified magnetic glassy carbon electrode; **CeO_2_ NPs:** Cerium oxide nanoparticles; **AgNPs:** Ag nanoparticles; **PPy@PEDOT:PSS:** polypyrrole@poly(3,4-ethylenedioxythiophene):polystyrene sulfonic acid; **SLT**: steel sheet; **N-HCSs:** Nitrogen-doped hollow carbon spheres**; CuMS:** Copper mesoporous**; NBC180:** Nanobiochar 180; **rGO/PPy NT:** nanocomposite of reduced graphene oxide/polypyrrole nanotubes; **POBI:** Porous organic polymers derivative; **CS:** Chitosan; ***p*-ADPA-4-ATP-Au:** Poly(4-aminodiphenylamine)-4-aminothiophenol-Au composite electrode; **Chitin:** Chitine; **GO:** Graphene oxide; **Co-Ce-NPs:** Cobalt-cerium nanoparticles; **IL:** ionic liquids**; NPG:** Nanoporous gold; **MWCNT:** multi-walled carbon nanotube; **MO:** Mineral oil; **Tl-Mn_3_O_4_/IL:** Thallium doped Mn nanostructures and ionic liquid (n-hexyl-3-methylimidazolium hexafluoro phosphate); **Pt-Pd NPs:** bimetallic Pt-Pd nanoparticles; **N-Gra:** nitrogen doped graphene; **PBIF:** fluorene-based,cross-linkedPICderivative; **PANI:** polyaniline; **AuNPs:** Gold nanoparticles; **GONRs:** Graphene oxide nanoribbons; **PPY-CHI:** polypyrrole-chitosan; **MTTiPNiH:** nickel hexacianoferrate (III); **MoS_2_:** molybdenum disulfide; **NF:** Nafion; **MgO:** oxide magnesium; **SWCNTs:** Single-Walled Carbon Nanotubes synthesized; **[Bmim][Tf2N]:** 1-Butyl-3-methylimidazolium bis(trifluoromethylsulfonyl)imide; **LaFeO_3_**: Nanoparticles of lanthanides orthoferrites; **TPyP:** 5,10,15,20-tetra(4-pyridyl)21*H*,23*H*-porphine; **TTiP:** titanium (IV) silsesquioxane and phosphoric acid; **ZTTiP:** HFAU zeolite; **ZTTiPNiH:** potassium hexacyanoferrate (III); **Pt:** Platimun electrode; **NiO-NPs:** Nickel oxide nanoparticles; **AF:** Acetylferrocene; **LuHCF:** Lutetium (III) hexacyanoferrate microparticles; **SSG-Au:** silicate sol–gel matrix nanostructures gold; **Si4Pic^+^Cl^—^:** 3-n-propyl(4 methylpyridinium) silsesquioxane chloride; **GCE:** Glassy carbon electrode; **CPE:** carbon paste electrode; **SPGE:** Screen printed graphite electrode; **SPCE:** Screen print carbon electrode; **GSPE:** Graphite screen printed electrode; **GC:** Glassy carbon electrode; **FTO:** fluorine-doped tin oxide; **µE:** gold microelectrode.

## 6. Electrochemical Sensors Using Amperometric Methodologies

Amperometric techniques represent a highly efficient alternative for sulfite quantification, particularly when high-throughput analysis and temporal resolution are required. Unlike voltammetric methods that sweep through a potential range, constant-potential amperometry involves applying a fixed oxidative overpotential to the working electrode, where the resulting steady-state current is directly proportional to the concentration of SO_3_^2−^ according to the Cottrell equation. This approach is particularly advantageous for its simplicity and its ability to provide real-time data, making it the preferred choice for integration into automated systems.

A significant trend in food quality control is the coupling of amperometric detection with FIA or HPLC. In these configurations, the modified electrode—often integrated into a thin-flow cell—acts as a highly sensitive detector for sulfite as it elutes from the carrier stream. The hydrodynamic conditions within these systems enhance mass transport to the electrode surface, effectively reducing the diffusion layer thickness and increasing the signal magnitude. Furthermore, the continuous flow of the carrier electrolyte serves to “wash” the electrode surface, mitigating the fouling effects typically encountered in static batch measurements of complex matrices like fruit juices or wastewater.

Despite its sensitivity, the selectivity of amperometry in complex food samples is highly dependent on the choice of the applied potential. To minimize the co-oxidation of common interferents such as L-ascorbic acid and phenolic antioxidants, researchers utilize electrodes modified with electrocatalytic materials, as can be seen in Table 3.

**Figure 9:**
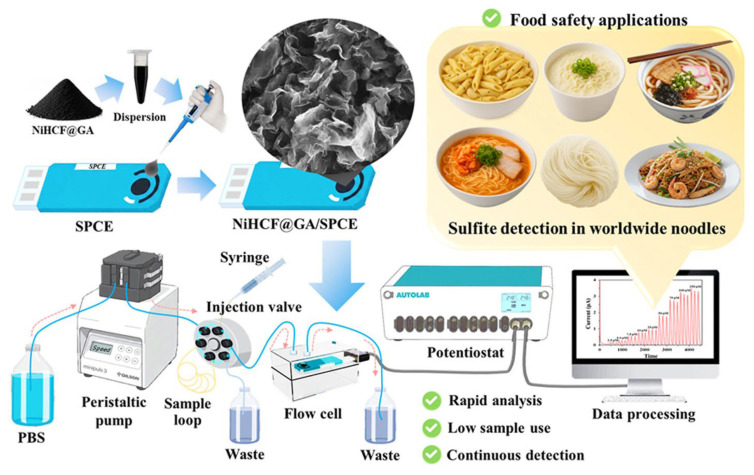
The fabrication of the proposed NiHCF@GA/SPCE, and the NiHCF@GA/SPCE used with the FIA system for continuous SO_3_^2−^ detection. Reproduced from [69].

Tuntiwongmetee et al. (2026) [69] reported a high-performance Flow Injection Amperometric (FIA-AMP) sensor using Nickel Hexacyanoferrate decorated on 3D Mesoporous Graphene Aerogel (NiHCF@GA), where show in Figure 9. The Substrate was a modified electrode integrated into an automated system for sulfite detection in worldwide noodles (Method Sample). The NiHCF@GA/SPCE sensor operated at 0.45 V, showing a linear range of 0.0025 to 27.50 mM and an LOD of 2.50 μM. Its accuracy was confirmed through the measurement of sulfites in worldwide noodle samples, yielding recoveries between 90.6% and 105.1%. The 3D aerogel structure provided an immense surface area and excellent conductivity, resulting in an exceptional Sensitivity and an ultra-low LOD. The study emphasized high repeatability and reproducibility through the automation of the FIA system. The use of a 3D graphene aerogel represents the pinnacle of carbon-based substrate evolution in this collection, significantly outperforming 2D rGO or MWCNT modifiers by minimizing mass transport limitations. However, the complexity of synthesizing 3D aerogels and the requirement for an FIA setup may limit its use to centralized high-throughput laboratories rather than point-of-care testing.

Gunasekaran and Govindarajan (2025) [63] presented a dual-format sensor using Chitosan-Imidazolium Ionic Liquid-MWCNT nanohybrids on both a GCE and a flexible bio-strip substrate. This modified electrode achieved a remarkably wide Linear range (70–20,200 µM) and an LOD of 1.83 µM using Amperometry. The Method Sample included food and environmental analysis. This work stands out by addressing the sustainability and flexibility of the sensor via the “bio-strip.” The synergistic use of Chitosan and IL ensures excellent film-forming properties and anti-fouling capabilities, leading to superior reproducibility. While its LOD is slightly higher than the 2026 aerogel-based study, its applicability in flexible, wearable, or portable electronics makes it a more versatile solution for modern field-monitoring needs.

Hernández-Saravia et al. (2024) [6] developed a sensor using copper(II) triazole complexes (Cu(L^NO2^)_2_) to modify a GCE. The Method employed was Chronoamperometry for the trace determination of sulfite in wine analysis. Using chronoamperometry at 0.90 V, the authors reported an LOD of 0.75 μM, an LOQ of 2.27 μM, a sensitivity of 7.43 × 10^−3^ μA μM^−1^, and a dynamic range from 10 to 5.00 μM. The sensor’s utility was demonstrated in the analysis of wine. The use of coordination complexes as modifiers offers a high degree of molecular precision compared to bulk nanoparticles. While the Sensitivity is adequate for wine standards, the molecular stability of the triazole complex under varying pH conditions and potential leaching of the complex from the electrode surface are critical factors that might compromise long-term repeatability and reproducibility compared to covalently anchored or nanostructured composites.

Pei and Wei (2024) [109] fabricated a NiCo_2_O_4_ decorated graphene composite on a GCE substrate. The modified electrode (NiCo_2_O_4_/rGO/GCE) was characterized by SEM and XRD, showing a synergistic effect that enhances sulfite oxidation current in amperometric measurements. The reported analytical parameters included a sensitivity of 0.32341 μA μM^−1^, a LOD of 3 nM, and an LOQ of 12 nM. The sensor was successfully applied for the accurate determination of sulfites in food samples. The sensor was optimized for food samples. The use of a bimetallic oxide (NiCo_2_O_4_) provides more active redox sites than monometallic oxides like CuO. This results in improved Sensitivity, though the dependence on a rigid GCE substrate and the drop-casting method for modification limits its reproducibility compared to the automated or flexible substrates seen in more recent years. It represents a solid evolution of transition metal oxide catalysts but lacks the structural innovation of 3D or flexible systems.

Massah et al. (2021) [59] proposed an amperometric sensor based on a metal–organic framework (MOF) for the sensitive determination of sulfite ions in the presence of ascorbic acid. The MIL-101(Cr)-CPE sensor was fabricated by modifying a carbon paste electrode with the MIL-101(Cr) MOF, which was synthesized via a hydrothermal route and characterized by high chemical stability and a large specific surface area. Electrochemical parameters obtained through SWV revealed a linear range of 2 to 70 μM and an LOD of 0.58 μM (S/N = 3). The sensor exhibited high selectivity, allowing for the simultaneous quantification of sulfite and ascorbic acid in commercial wine samples.

Wang et al. (2019) [110] developed highly sensitive and selective sulfite sensors based on solution-gated graphene transistors (SGGTs) functionalized with multi-walled carbon nanotube (MWCNT) gate electrodes. The synthesis methodology leveraged gate electrode functionalization to significantly enhance charge transfer and selectivity. The device proved highly effective for detecting sulfites in complex liquor samples, providing a low-cost and rapid alternative to conventional analytical techniques.

**Figure 10:**
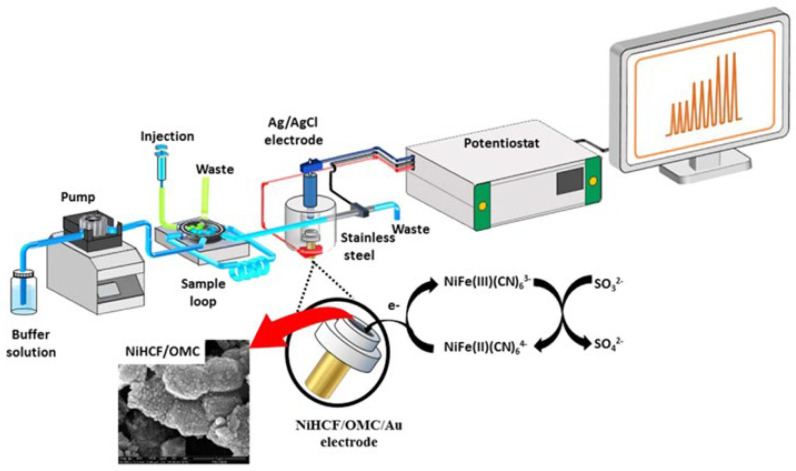
Schematic diagram of the flow injection amperometric sensor system. The oxidation current of sulfite was obtained from the oxidation current of NiHCF as the electrocatalytic mediator. Reproduced from [77].

Preecharueangrit et al. (2018) [77] fabricated an amperometric sensor through the electrodeposition of nickel hexacyanoferrate (NiHCF) onto a layer of ordered mesoporous carbon (OMC) coating a gold electrode (NiHCF/OMC/Au). This architecture facilitated high electrocatalytic activity toward sulfite oxidation under flow conditions. Integrated into a flow injection analysis (FIA) system see in Figure 10, the sensor exhibited an extensive linear range from 2.5 μM to 50 mM and an LOD of 2.5 μM. The method was applied to determine sulfite levels in noodle products, including vermicelli, instant noodles, and macaroni.

Devaramani et al. (2017) [73] proposed a robust modified electrode based on covalent anchoring of cobalt hexacyanoferrate (CoHCF) on graphitic carbon. The material underwent structural characterization via XRD and FTIR prior to the fabrication of a robust and renewable pellet electrode. The proposed sensor demonstrated a linear range of 4 to 128 μM, an LOD of 1.7 μM, and a LOQ of 5.8 μM. The practical applicability was verified in various food sample matrices (Tomato Ketchup, Wine, Jam, Sugar and Dry grapes), showing high precision compared to the iodometric method. The covalent anchoring strategy significantly enhances mechanical stability, leading to excellent repeatability compared to simple drop-casting. However, the pellet electrode format is less adaptable for miniaturization than the SPE formats that became dominant in later years.

**Table 3:** Electrode materials for sulfite detection by amperometry: fabrication method, analysed sample, and analytical features (linear range, LOD, LOQ and sensitivity).

Electrode/Substrate	Method	Sample	Linear Range (μM)	LOD (μM)	LOQ (μM)	Sensitivity	Ref.
polyCoFeRu/GCE	Solvothermal	Tap water, rain water and river water	6.6–178.17	0.06	0.20	6.98 μA mM^−1^	[62]
NiHCF@GA/SPCE	Hydrothermal	Macaroni, vermicelli, spaghetti, Pad Thai, white Chinese noodles, udon, ramyeon, and instant noodles	2.50–27,500	2.50	—	9.22 ± 0.14 μA mM^−1^	[69]
Cs-IL/MWCNT@GCE	Solvothermal	Raisins and apricots	70–20,200	1.83	6.08	10.5 μA mM^−1^	[63]
Cu(^LNO2^)_2_/GCE	Solvothermal	White wine and red wine	10.00–5000	0.75	2.27	7.43 × 10−3 μA μM^−1^	[6]
AuNPs-GCE	Hydrothermal	Tap water	6.66–1020	0.41	—	2.46 × 10−7 μA μM^−1^	[111]
NiCo_2_O_4_/rGO/GCE	Hydrothermal	Water, grapes, apple juice	5–4850	3 nM	12 nM	0.32341 μA μM^−1^	[109]
Sm_1.9_Ce_0.1_CuO_4_	Hydrothermal	—	10–100	—	—	36.6 mA cm^−1^ M^−1^	[112]
CuO/rGO/SPE	Hydrothermal	Anise aqueous	1–300	0.2425	—	122.3 μA cm^−1^ μM^−1^	[113]
SPE	—	White wine and red wine	6–200 mg L^−1^	2.38 mg L^−1^	7.5 mg L^−1^	—	[114]
PTZ-IL/MWCNT/GCE	Solvothermal	Vinegar and pickle water	30–1177	9.3	—	282.2 μA cm^−1^ mM^−1^	[115]
MIL-101(Cr)-CPE	Solvothermal	White wine	2–70	0.58	—	0.095 μA μM^−1^	[59]
Pt-NGC electrode	Electrodeposition	—	80–5000	3	—	—	[75]
PB-PPy 1:1:10	Solvothermal	Red wine and rosé wine	0–5 mM	—	—	—	[116]
Pd-CNF/CP	Hydrothermal	—	120–600 ppm	10 ppm	—	4.75 μA cm^−1^ ppm^−1^	[64]
CTAB/MWCNT/Au	Sonication	Vodka and Laobaigan	1–10	30 nM	—	—	[110]
Ni/poly(4-AB)/SDS/CPE	Solvothermal	Weak liquor	0.1–1	0.063	—	0.0341 μA μM^−1^	[117]
NiO nanoplatelet/GCE	Hydrothermal	Water samples	16.2–600	8.8	—	2.8 μA cm^−1^ mM^−1^	[118]
OMC/NiHCF/Au	Electrodeposition/Solvothermal	Vermicelli, instant noodles, noodles, and macaroni	0.0025–50 mM	2.5	—	6.57 μA mM^−1^	[77]
C@NiCo_2_O_4_	Hydrothermal	—	0.3–724	0.05	—	3.57 μA cm^−1^ mM^−1^	[119]
CoHCF-BLPE	Chemical modification	Tomato ketchup, wine, jam, sugar and dry grapes	4–128	1.74	5.8	0.008 μA μM^−1^	[73]
APS-Au NPs	Chemical modification	—	100 nM–150 μM	100 nM	—	0.001 nA nM^−1^	[120]
CNT electrode	—	Dried fruits and lemon juice	500–6000 ppm	4 ppm	—	—	[121]

**polyCoFeRu:** Ru(II), Co(II), and Fe(II) coordinated with a bister-pyridine ligand; **NiHCF@GA:** composite of nickel hexacyanoferrate on 3D mesoporous graphene aerogel; **IL:** ionic liquids**; MWCNT:** multi-walled carbon nanotube; **Cs:** Chitosan; **Cu(L^NO2^)_2_:** copper (II) 5-methyl-1-(4-nitrophenyl)-1H-1,2,3-triazole-4-carboxylate; **AuNPs:** Gold nanoparticles; **rGO:** Reduce graphene oxide; **NiC_2_O_4_:** Nickel cobaltite nanoparticles; **CuO:** copper oxide; **PTZ:** Phenothiazine; **Pt-NGC:** Pt particles electrodeposited on the nitrogen-containing functional groups; **PB-PPy 1:1:10:** Prussian blue-polypyrrole; **Pd-CNF:** modified carbon nanofiber; **CTAB:** cetyltrimethylammonium bromide; **Ni/poly(4-AB)/SDS:** Ni/poly(4-aminobenzoic acid)/sodium dodecylsulfate; **NiCHF:** Nickel hexacyanoferrate; **OMC:** Mesoporous carbon covering; **C@NiCo_2_O_4_:** Carbon coated nickel cobaltite nanoparticles; **CoHCF:** Cobalt hexacyanoferrate; **BLPE:** Binderless pellet; **APS:** 3-(aminopropyl)triethoxysilane; **CNT:** Carbon nanotube; **[Fe(tacn)_2_]^3+^:** 1,2-bis(1,4,7-triaza-1-cyclononyl)ethane iron(III) ([Fe(dtne)]^3+^); **GCE:** Glassy carbon electrode; **SPCE:** Screen print carbon electrode; **SPE:** Screen print electrode; **CPE:** Carbon paste electrode; **Au:** Gold.

## 7. Electrochemical Sensors Using Square Wave Voltammetry Methodology

Beyond the capabilities of DPV, Square Wave Voltammetry (SWV) has emerged as an exceptionally powerful technique for the rapid quantification of sulfite in complex matrices. The fundamental advantage of SWV lies in its unique waveform—a symmetrical square wave superimposed on a staircase potential—which allows for significantly higher scanning speeds compared to other pulse techniques. This rapid execution minimizes the time the electrode is held at high oxidizing potentials, thereby reducing the risk of electrode fouling caused by the irreversible adsorption of wine proteins or polyphenolic oxidation products.

In the context of sulfite sensing, SWV provides a sophisticated level of background suppression, can see in Table 4. By recording both the forward (oxidative) and reverse (reductive) currents, the resulting net current (∆i) enhances the sensitivity and peak resolution. This feature is particularly critical when analyzing beverages with dense chemical profiles, where the SO_3_^2−^ signal may be obscured by baseline drift or proximal interferents. The kinetic sensitivity of SWV also allows for the detection of subtle changes in the electron transfer rate, providing a robust analytical response even at trace levels (<10 mg L^−1^), which is vital for verifying compliance with international food labeling regulations.

The integration of SWV with disposable modified electrodes—such as SPCEs decorated with metal nanoparticles—represents the current trend toward “point-of-need” testing. Because SWV can complete a full analytical scan in seconds, it is ideally suited for integration into portable sensing platforms for real-time monitoring of sulfite during various stages of food processing or in wastewater management.

**Figure 11:**
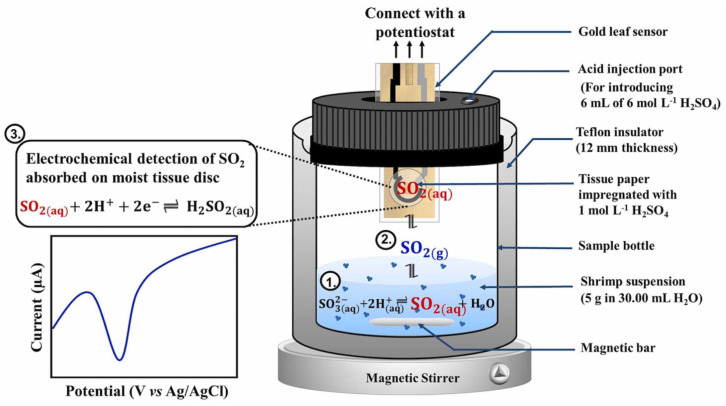
Schematics of the setup for the direct quantitation of sulfite residue in frozen shrimp by in situ electrochemical detection in a gas medium of SO_2_ adsorbed on moist tissue paper. Reproduced from [58].

Jantawong et al. (2023) [58] developed an unconventional electrochemical sensor for the in situ detection of sulfite residue in frozen shrimp samples by quantifying SO_2_ gas. The device features a suspended gold leaf electrode unit where a facial tissue paper, moistened with a supporting electrolyte (0.1 M H_2_SO_4_), serves as both the porous substrate and the medium for gas absorption (see Figure 11). The modified electrode system effectively creates a liquid layer within the paper’s pores to facilitate voltammetric detection in a gas medium. Using SWV after acidification of the shrimp tissue to generate gas, the sensor achieved a linear range of 3 to 240 mg kg^−1^ SO_2_ and a LOD of 2 mg kg^−1^ SO_2_. This approach bypasses the typical matrix interferences found in aqueous food extracts by transferring the analyte to the gas phase, a significant advantage over traditional liquid-phase sensors. While the sensitivity is sufficient for regulatory compliance (100 mg kg^−1^ limit), the reliance on a manual gas generation step might introduce variability in repeatability compared to fully automated systems, although the authors emphasize its low cost (0.2 USD) and reusability for up to 3 months as key factors for point-of-need applicability.

Araújo et al. (2021) [60] introduced a miniaturized electroanalytical device integrated with a headspace-like gas extraction unit for determining sulfite in beverages. The sensor utilizes a copper electrode as the working element, integrated into a cell fabricated from alternative, low-cost materials. The method involves a single-step gas extraction and SWV detection, significantly simplifying the sample pretreatment usually required for complex matrices. The system demonstrated a linear range between 10 and 150 μM (based on provided data trends) and achieved a LOD of 4.0 μM after a 10 min extraction period. An analysis of this work reveals that the integration of sample pretreatment (gas diffusion) directly into the miniaturized cell not only improves selectivity by isolating the volatile SO_2_ but also addresses the dilution issues common in conventional cells. The study provides high temporal resolution for real-time extraction curves, which is a superior feature for optimizing kinetics compared to the static headspace methods often seen in earlier literature.

Martins et al. (2019) [122] proposed a laser-pyrolyzed electrochemical paper-based analytical device (LP-ePAD) for sulfite analysis in commercial beverages. The electrode is made of conductive carbon material produced by CO_2_ laser pyrolysis of a paperboard substrate, which serves as a low-cost and reproducible platform. This sensor was coupled with a gas-diffusion microextraction (GDME) unit, utilizing SWV as the detection method. The device exhibited a LOD of 1 mg L^−1^ and demonstrated excellent repeatability and reproducibility, with a coefficient of variation (CV) below 7%. From a critical perspective, the use of laser pyrolysis represents a major step forward in ePAD fabrication, eliminating the impurities and manual inconsistencies associated with traditional screen-printing or drawing methods. The integration with GDME ensures that the paper-based electrode is protected from direct contact with the complex sample matrix, thereby extending the device’s life and maintaining high sensitivity in “real-world” applications.

Winiarski et al. (2017) [123] reported the electrochemical reduction of sulfite using a carbon paste electrode (CPE) modified with AuNPs and Si4Pic^+^Cl^−^. The Au-Si4Pic^+^Cl^−^/CPE utilized Si4Pic^+^Cl^−^ as a stabilizing agent for the 45 nm AuNPs. The method employed was SWV in an acidic medium, where sulfite exists as electroactive SO_2_, showing a reduction peak at ca. −0.35 V. The linear range was 2.54 to 48.6 mg L^−1^, with an LOD and LOQ of 0.88 and 2.68 mg L^−1^, respectively. The sensor was validated in white wine and coconut water, showing no matrix effects. This work is distinctive because it focuses on the reduction of sulfite rather than oxidation, which can be advantageous in avoiding common oxidizable interferences in food. However, the LOD is significantly higher than that reported for AuNP-graphene composites, suggesting that while silsesquioxane provides stability, it may not offer the same electronic enhancement as graphene-based substrates.

Xu et al. (2016) [124] developed a simple electrochemical sensor based on a Carbon Black Paste Electrode (CBE) for determining sulfites in rice wine. The electrode consists of a mixture of carbon black powder and liquid paraffin (2:1 ratio) packed into a cavity without further modification. The method employed first-order derivative SWV, which sharpened the oxidation peaks and significantly enhanced the sensitivity compared to bare glassy carbon or graphite electrodes. The sensor provided a linear range of 0.008 to 1.0 mM and a LOD of 6.0 μM. The practical applicability of the device was successfully validated in rice wine samples. This work demonstrates that high-performance sensing can be achieved using relatively inexpensive carbon black instead of costlier nanomaterials like carbon nanotubes or graphene. While the derivative processing helps resolve the peaks, the sensor’s selectivity in the presence of other common wine antioxidants (like polyphenols) is less robust than the gas-diffusion or MOF-modified systems seen in later years, suggesting that this solution is best suited for less complex or pre-filtered samples.

**Table 4:** Electrode materials for sulfite detection by square wave voltammetry: fabrication method, analysed sample, and analytical features (linear range, LOD, LOQ and sensitivity).

Electrode/Substrate	Method	Sample	Linear Range (μM)	LOD (μM)	LOQ (μM)	Sensitivity	Ref.
gold leaf sensor	—	Spiked shrimp	3–240 mg kg^−1^	2 mg kg^−1^	—	—	[58]
Cu electrode	—	Tap water, grape juice, and coconut water	20–100	4.0	13.0	0.095 μA μM^−1^	[60]
GDME-LP-ePAD	—	Wine, Beer, No-alcoholic beer, vinegar and coconut water	2.5–25 mg L^−1^	1.2 mg L^−1^	3.9 mg L^−1^	—	[122]
SPCE	—	Beer	0.1 —1 mg L^−1^	0.13 mg L^−1^	0.050 mg L^−1^	4.33 nA mg^−1^ L	[125]
CuSPE	—	Tap water	0.1–1.0 mM	41	—	2832.3 μA cm^−2^ mM^−1^	[126]
CBE	—	Rice Wine	0.008–1.0 mM	6	—	132.84 × 10^−3^ μA M^−1^	[124]
CPE/Au-Si4Pic^+^Cl^−^	Hydrothermal	Coconut water and wine	2.54–48.6 mg L^−1^	0.88 mg L^−1^	2.68 mg L^−1^	0.353 μA mg^−1^ L	[123]

**MIL-101(Cr)**: metal–organic framework; **Cu electrode:** Copper electrode; **LP-ePAD:** Laser-pyrolyzed electrochemical paper-based analytical device; **GDME:** gas-diffusion microextraction; **CuSPE:** copper screen-printed electrodes; **CBE:** carbon black paste electrode; **CPE:** Carbon paste electrode; **SPCE:** Screen print carbon electrode; **Si4Pic^+^Cl^−^:** 3-n-propyl(4 methylpyridinium) silsesquioxane chloride; **GCE:** Glassy carbon electrode.

## 8. Biosensors

The integration of nanozymes—nanostructured materials with intrinsic enzyme-like activities—has emerged as a transformative strategy to overcome the limitations of protein-based biosensors. Unlike natural Sulfite Oxidase (SOx), which is highly specific but suffers from low operational stability and complex immobilization requirements, nanozymes offer superior chemical robustness, ease of mass production, and resistance to harsh environmental conditions such as the high ethanol content or acidic pH typical of viticultural matrices.

Nanozymes for sulfite sensing are typically synthesized through hydrothermal, sol–gel, or electrochemical deposition methods to achieve precise control over surface area and active site exposure. For instance, metal-oxide-based nanozymes, such as Ce^3+^-doped CuO or LaFeO_3_ nanoparticles, leverage oxygen vacancies and transition metal redox couples to mimic the catalytic center of SOx. The catalytic mechanism generally involves the facilitated adsorption of the SO_3_^2−^ anion onto the nanostructured surface, followed by a multi-step electron transfer that lowers the activation energy for oxidation to SO_4_^2−^. In bimetallic systems, such as Pt-Pd nanoparticles, a synergistic effect is observed where one metal facilitates analyte adsorption while the other promotes rapid charge transfer, significantly enhancing the electrocatalytic current.

While natural SOx enzymes exhibit unparalleled substrate specificity, they are frequently limited by long-range electron tunneling distances due to the deep embedding of the Mo-cofactor within the protein scaffold. In contrast, nanozymes function as “third-generation” catalysts by acting as both the recognition element and the electrical wire, ensuring direct and rapid electron transfer to the electrode. Although nanozymes may encounter challenges regarding cross-sensitivity with other reductants (e.g., ascorbic acid), their ability to maintain catalytic activity over extended storage periods and across wide temperature ranges provides a clear industrial advantage over traditional enzymatic assays.

Recent applications demonstrate the versatility of nanozymes in complex food systems [127]. For example, acid-treated Fe_3_O_4_@SiO_2_ magnetic nanoparticles have been successfully deployed for the detection of sulfite in milk, achieving detection limits in the low micromolar range while maintaining robustness against protein interference. Similarly, La^3+^-doped Co_3_O_4_ nanocubes and gold-nanoparticle-decorated carbon nanotubes (AuNPs-CNTs) have shown high recovery rates (97–103%) in wine and beverage samples. These “artificial enzymes” are increasingly prioritized for the development of portable, field-deployable sensors, bridging the gap between high-precision laboratory analysis and real-time industrial quality control.

A critical challenge in biosensor design for food analysis is the achievement of efficient direct electron transfer between the enzyme’s redox center and the electrode surface, see Table 5. Due to the deep embedding of the Mo-cofactor within the protein scaffold, long-range tunneling distances often hinder kinetic performance. To overcome this, researchers employ “third-generation” biosensor architectures utilizing nanomaterials—such as carbon nanotubes, graphene, or gold nanoparticles—and redox mediators (e.g., tetrathiafulvalene or ferrocene derivatives). These materials act as “electrical wires,” facilitating rapid charge transport and allowing the biosensor to operate at lower overpotentials, which is crucial for preventing the co-oxidation of polyphenols in enological samples.

The practical utility of sulfite biosensors in the food industry is often dictated by the immobilization strategy used to attach the enzyme to the transducer. Techniques such as covalent cross-linking with glutaraldehyde, entrapment within biocompatible Nafion or chitosan membranes, and physical adsorption onto nanostructured interfaces are rigorously evaluated to maximize enzymatic activity and operational stability. While biosensors offer unparalleled selectivity—capable of detecting sulfite in the presence of high concentrations of ascorbic acid without complex pre-treatment—their commercial application must address the inherent sensitivity of proteins to the extreme pH values and ethanol content often found in real food matrices and beverages.

Hussain and Adeloju (2023) [128] developed an ultrasensitive amperometric biosensor by biofunctionalizing a polypyrrole nanowire array (PPyNWA) integrated with platinum nanoparticles (PtNPs) on a platinum substrate. The modified electrode (PPyNWA-PtNPs-SOx) was fabricated using a dual-step anodization method with an anodic aluminum oxide (AAO) template for the nanowires, followed by potential cycling for PtNP deposition and adsorption of sulfite oxidase (SOx). Using amperometry at a current density of 0.7 mA cm^−2^, the sensor was tested in beer and wine samples, achieving a linear range of 0.12 to 1200 µM, a remarkably low LOD of 12.35 nM, and a sensitivity of 7.33 μA cm^−2^ mM^−1^. The study demonstrates excellent repeatability and reproducibility through recovery efficiencies of 97–103% in real matrices. The use of a nanowire array significantly surpasses traditional planar electrodes by providing a larger surface area and superior conduction channels, which directly correlates to the ultra-low LOD compared to earlier works. However, the complexity of the AAO template fabrication and the 8 h adsorption time for the enzyme might limit its high-throughput industrial scalability compared to simpler paste-based electrodes.

Sroysee et al. (2021) [23] reported a high-performance biosensor utilizing a 3D rGO aerogel decorated with AgNPs as the substrate. The modified electrode (rGO@Ag-Cys-FA-SOx) involved functionalization with cysteine (Cys) and folic acid (FA) to anchor the SOx enzyme via stable thiol bonding. Using a continuous flow injection system (FI-amperometry), the sensor analyzed canned fruit products, showing high bio-electrocatalytic activity and a fast electron transfer rate. The 3D architecture of the aerogel provides a high specific pore volume that enhances enzyme loading and stability. A comparison suggests that while AgNPs provide excellent conductivity and a stable anchoring site, the rGO aerogel’s mechanical fragility in flow systems requires careful housing (like the CamorHyde 1000A-PA) compared to more robust metallic or carbon-paste substrates. The inclusion of FA and Cys as a linker shell represents a strategic approach to maintaining enzyme bioactivity that is more sophisticated than simple entrapment.

**Figure 12:**
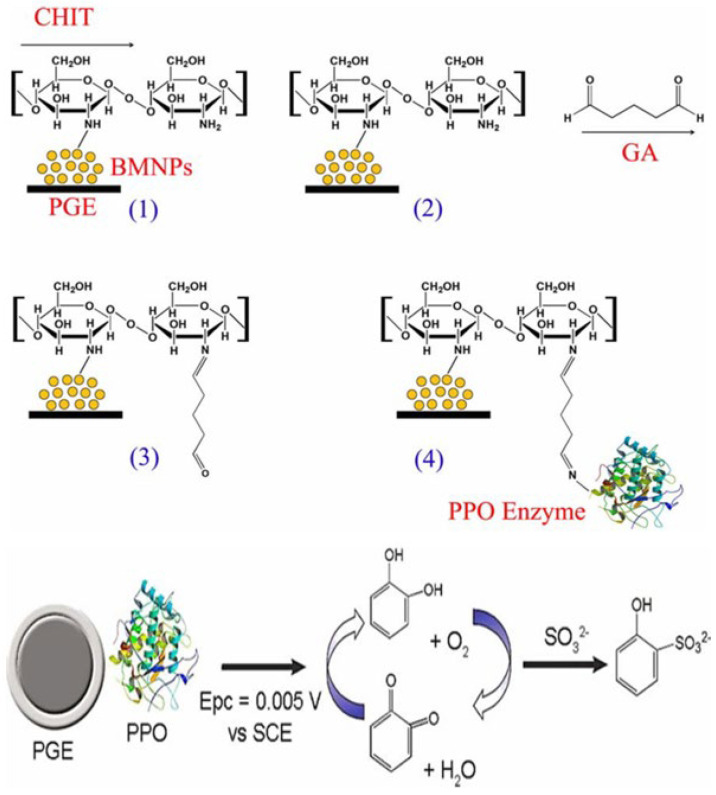
Schematic illustration of the different steps for the construction of a sulfite biosensor. (1) CHIT binds to BMNPs through electrostatic interactions; (2) amine-aldehyde bond between CHIT and GA; (3) aldehyde-amine bond between GA and PPO enzyme; (4) the PGE/BMNPs/CHIT/GA/PPO biosensor is obtained. Reproduced from [58].

Teixeira et al. (2020) [129] proposed an indirect electrochemical biosensor based on babassu mesocarp nanoparticles (BMNPs) immobilized on a pyrolytic graphite electrode (PGE). The modified electrode (PGE/BMNPs/CHIT/GA/PPO) used chitosan (CHIT) and glutaraldehyde (GA) to support polyphenol oxidase (PPO) obtained from sweet potatoes (see Figure 12). The method relied on the inhibitory effect of sulfite on PPO activity, measured via SWV and CV. Applied to industrial juices, the sensor reached a linear range (noted via sensitivity) with an LOD of 0.151 µM, a LOQ of 0.452 µM, and a sensitivity of 2.18 µA µM^−1^. This work stands out for using a natural, non-toxic polymer (babassu) as a binder, offering a sustainable alternative to synthetic matrices. However, as an indirect method based on inhibition, it may face competition from other inhibitors present in complex juice matrices, potentially affecting selectivity compared to the SOx-based direct oxidation sensors.

Adeloju and Hussain (2016) [129] developed a potentiometric biosensor by entrapping SOx within an ultrathin PPy film on a Pt electrode modified with PtNPs. The modified electrode (PtNPs/PPy-SOx) was prepared by cycling the potential to deposit 30–40 nm PtNPs, followed by one-step electropolymerization. Tested in wine and beer, the sensor showed a linear range of 0.75 to 65 µM, an LOD of 12.4 nM, and a sensitivity of 57.5 mV dec^−1^. The response time was 3–5 s. A critical analysis reveals that this potentiometric approach offers simplicity and a lower LOD than many contemporary amperometric sensors, likely due to the high surface-to-volume ratio of the PtNPs. However, the linear range is significantly narrower than the 2023 NWA-based version by the same authors, highlighting how nanostructuring (nanowires vs. nanoparticles) can drastically extend the analytical utility.

Sroysee et al. (2016) [127] described an online amperometric biosensor using SOx immobilized on a magnetite-gold-folate nanocomposite. The substrate was a CPE, and the modified electrode Fe_3_O_4_@Au-Cys-FA /CPE) used a PDMS/mineral oil binder for enhanced stability. Using FI-amperometry at +0.35 V, the system analyzed wines and pickled foods, achieving a linear range of 0.1–200 mg L^−1^, an LOD of 10 µg L^−1^, and a sensitivity evidenced by a rapid sample throughput of 109 samples h^−1^. The repeatability was confirmed with an RSD of 3.1, and stability lasted for 2 weeks. Critically, the use of magnetic nanoparticles (Fe_3_O_4_) allows for potential magnetic separation or easier electrode renewal, and the low operating potential (+0.35 V vs. Ag/AgCl) minimizes interference from common antioxidants like ascorbic acid, a significant advantage over non-enzymatic sensors.

Kalimuthu et al. (2016) [130] demonstrated the mediated electrocatalytic voltammetry of human sulfite oxidase (HSO) using a GCE as the Substrate. The modified electrode incorporated synthetic iron(III) complexes, specifically bis(1,4,7-triazacyclononane)iron(III) ([Fe(tacn)_2_]^3+^) and 1,2-bis(1,4,7-triaza-1-cyclononyl)ethane iron(III) ([Fe(dtne)]^3+^), as electron acceptors to facilitate the enzymatic reaction. Using Amperometry and CV as the Method, the study focused on the kinetic parameters of the HSO/mediator system. The sensor exhibited a linear response range from 5.0 × 10^−6^ to 8.0 × 10^−4^ M, with a LOD of 0.2 μM (S/N = 3). This enzymatic approach offers superior selectivity compared to non-enzymatic sensors; however, the stability of the HSO enzyme on the GCE surface remains a significant bottleneck for long-term repeatability and reproducibility. Compared to later nanoparticle-based sensors, this method operates at much lower overpotentials but is constrained by the inherent fragility of biological components and a more complex immobilization process.

**Table 5:** Electrode materials for sulphite detection by biosensors: fabrication method, analysed sample, and analytical features (linear range, LOD, LOQ and sensitivity).

Electrode/Substrate	Method	Sample	Linear Range (μM)	LOD (μM)	LOQ (μM)	Sensitivity	Ref.
PPyNWA-PtNPs-SOx	Electrochemical	Beer and wine	0.12–1200	12.35 nM	—	7.33 μA cm^−2^ mM^−1^	[128]
rGO@Ag-Cys-FA-SOx/GCE	Chemical synthesis/Hydrothermal	Lychee, rambutan, and longan	0.025–40 mg L^−1^	0.007 mg L^−1^	—	8.775 μA mg^−1^ L	[23]
PGE/BMNPs/CHIT/GA/PPO^IX^	Solthermal	Juice sample	5–80	0.151	0.452	2.18 μA μM^−1^	[129]
*h*SO/APTES/ITO	—	—	2–20	0.5	—	—	[131]
PtNPs/PPy-SOx	Electrochemical	Wine and Beer	0.75–65	12.4 nM	—	57.5 mV dec^−1^	[70]
Fe_3_O_4_@Au-Cys-FA/CPE	Hydrothermal	Wine, ginger, mango and cabbage	0.1–200 mg L^−1^	10 μg L^−1^	—	1.086 μA mg^−1^ L	[127]
[Fe(tacn)_2_]^3+^	Solvothermal	Wine and Beer	10–180	0.2 pM	—	1.202 nA μM^−1^	[130]

**PPyNWA:** polypyrrole nanowire array; **PtNPs:** platinum-nanoparticle-modified; **SOx:** sulfite oxidaseenzyme; **rGO:** Reduce graphene oxide; **AgNPs:** Silver nanoparticles; **Cys:** Cysteine; **FA:** Folic acid; **PGE:** Pyrolytic graphite electrode; **BMNPs:** Babassu mesocarp nanoparticles; **CHIT:** immobilization of chitosan; **GA:** Glutaraldehyde; **PPO^IX^:** Polyphenol oxidase; ***h*SO:** human sulfite oxidase; **APTES:** Aminopropyltriethoxysilane; **PPy:** polypyrrole; **Fe_3_O_4_@Au-Cys-FA**: folic acid-cysteine-conjugated gold-coated magnetite core shell; **ITO:** indium tin oxide; **GCE:** Glassy carbon electrode; **SPE:** screen printed electrode.

## 9. Future Perspectives

The evolution of electrochemical sulfite sensing is currently at a technological crossroads, transitioning from proof-of-concept laboratory prototypes to integrated analytical platforms capable of operating within the rigors of industrial food processing. Despite the significant advancements in nanomaterial-assisted electrocatalysis and enzymatic specificity discussed herein, several critical challenges must be addressed to ensure the widespread adoption of these technologies. One of the primary frontiers in this field is the enhancement of sensor selectivity in “recalcitrant” matrices, such as red wines and highly processed meats, where the high concentration of polyphenols and organic acids frequently leads to overlapping faradaic signals. Future research should prioritize the development of multi-layered electrode architectures that combine size-exclusion membranes with high-affinity electrocatalysts, such as transition metal hexacyanoferrates or bimetallic nanoparticles, to effectively decouple the sulfite response from endogenous interferents.

Furthermore, the “point-of-use” paradigm demands the miniaturization and cost-reduction in transducers. The success of SPE technologies provides a robust framework for creating disposable, mass-produced sensors. However, the future lies in the integration of these sensors with smartphone-based potentiostats and Internet of Things (IoT) frameworks. This would allow for real-time, decentralized monitoring of sulfite levels across the entire supply chain, from the production line to the retail shelf, democratizing high-precision analysis for small-scale producers who currently lack access to expensive instrumental methods like HPLC or ion chromatography (IC). Regarding biosensing, the stabilization of SOx remains a bottleneck for commercial viability. Future perspectives should focus on the use of advanced immobilization matrices—such as MOFs or carbon-based nanocomposites—to preserve enzymatic activity under the varying pH and ethanol conditions typical of viticultural and beverage samples. Additionally, the exploration of “artificial enzymes” or nanozymes, which mimic the catalytic center of SOx but offer superior chemical robustness, represents a promising avenue for developing the next generation of non-enzymatic sulfite sensors.

Finally, a systematic shift toward “green” analytical chemistry is anticipated. Future methodologies must minimize the use of toxic reagents and emphasize the fabrication of electrodes using sustainable materials, such as bio-char or chitosan-derived nanocomposites, ensuring that the monitoring of food safety does not come at the cost of environmental integrity. By bridging the gap between fundamental electrochemistry and industrial engineering, these advancements will ultimately ensure a safer food supply and stricter compliance with international regulatory frameworks.

## 10. Conclusions

The analytical surveillance of sulfite species (SO_3_^2−^, SO_2_ and HSO_3_^−^) in the agri-food sector is at a critical technological crossroads. While this review has demonstrated that the integration of nanostructured interfaces—ranging from 1D/2D hybrid nanomaterials like rGO/PPy nanotubes to bimetallic Pt-Pd systems—has successfully pushed the LOD to the nanomolar range, a significant “translational gap” persists between laboratory-scale prototypes and robust industrial applications. The superior electrocatalytic performance observed in controlled aqueous media often degrades when confronted with the inherent chemo-diversity of food matrices. Specifically, the competitive adsorption of polyphenols and organic acids on modified surfaces leads to chronic electrode fouling and signal attenuation, which remains the primary bottleneck for the long-term stability of non-enzymatic sensors.

Future research must shift from the mere optimization of peak currents toward the engineering of “matrix-resilient” architectures. This involves the strategic implementation of multi-layered designs where size-exclusion or charge-selective membranes (e.g., Nafion, chitosan, or molecularly imprinted polymers) are coupled with high-affinity electrocatalysts to decouple the sulfite response from endogenous antioxidants like L-ascorbic acid. Furthermore, while nanozymes offer a robust alternative to unstable protein-based biosensors, their substrate specificity must be rigorously validated against “recalcitrant” matrices such as red wine or processed meats. The transition to decentralized, “point-of-use” testing—facilitated by SPE technologies and smartphone-integrated potentiostats—represents the most viable pathway for democratizing high-precision analysis in the food industry.

Finally, the alignment with “green” analytical chemistry is no longer optional. The development of sensors utilizing bio-derived substrates, such as bio-char or chitin-nanocomposites, ensures that food safety monitoring protocols remain environmentally sustainable. By bridging fundamental electrochemistry with materials science and portable engineering, the next generation of electrochemical sensors will move beyond academic curiosity to become indispensable tools for real-time quality control and regulatory compliance in the global food supply chain.

## Figures and Tables

**Table 1:** Comparative analysis of analytical methods.

Feature	Monier-Williams	HPLC	Electrochemical Methods
Principle	Acid-base titration following distillation.	Affinity-based separation with UV/FLD detection.	Redox reaction at the electrode interface.
Analysis time	High (60–120 min per sample).	Moderate (20–45 min).	Rapid/Real-time (<5 min).
Recovery rate	80–90% (subject to volatile loss).	90–105% (highly accurate).	95–102% (highly efficient).
Precision (RSD)	Low precision (RSD > 10%).	High precision (RSD < 3%).	High precision (RSD 3–5%).
LOD	~10 ppm (standard).	Trace levels (ppb range).	Low ppm to ppb range.
Operational cost	Low (common glass/reagents).	High (solvents, columns, maintenance).	Very Low (minimal reagent use).
Portability	Non-portable (bench-top setup).	Non-portable (heavy instrumentation).	Field-deployable (handheld devices).
Sample preparation	Labor-intensive (acid digestion).	Complex (filtration/derivatization).	Minimal/Direct injection.
Main Limitations	Time-consuming; prone to analyte loss during distillation.	High maintenance; requires extensive sample cleanup.	Electrode fouling; interference from co-existing antioxidants.
Ref.	[7]	[76]	[77]

## Data Availability

Data are contained within the article.
